# Association among blood pressure, end-tidal carbon dioxide, peripheral oxygen saturation and mortality in prehospital post-resuscitation care

**DOI:** 10.1016/j.resplu.2024.100577

**Published:** 2024-02-13

**Authors:** Elina Heikkilä, Piritta Setälä, Milla Jousi, Jouni Nurmi

**Affiliations:** a Faculty of Medicine, University of Helsinki, Helsinki, Finland; b Emergency Medical Services, Centre for Prehospital Emergency Care, Tampere University Hospital, Tampere, Finland; c Emergency Medicine and Services, Helsinki University Hospital and University of Helsinki, Finland; d FinnHEMS Research and Development Unit, Finland 4

**Keywords:** Helicopter emergency medical service, Emergency medical service, Critical care, Postresuscitation, Prehospital care

## Abstract

**Aim:**

Post-resuscitation care is described as the fourth link in a chain of survival in resuscitation guidelines. However, data on prehospital post-resuscitation care is scarce. We aimed to examine the association among systolic blood pressure (SBP), peripheral oxygen saturation (SpO_2_) and end-tidal carbon dioxide (EtCO_2_) after prehospital stabilisation and outcome among patients resuscitated from out-of-hospital cardiac arrest (OHCA).

**Methods:**

In this retrospective study, we evaluated association of the last measured prehospital SBP, SpO_2_ and EtCO_2_ before patient handover with 30-day and one-year mortality in 2,611 patients receiving prehospital post-resuscitation care by helicopter emergency medical services in Finland. Statistical analyses were completed through locally estimated scatterplot smoothing (LOESS) and multivariable logistic regression. The regression analyses were adjusted by sex, age, initial rhythm, bystander CPR, and time interval from collapse to the return of spontaneous circulation (ROSC).

**Results:**

Mortality related to SBP and EtCO_2_ values were U-shaped and lowest at 135 mmHg and 4.7 kPa, respectively, whereas higher SpO2 shifted towards lower mortality. In adjusted analyses, increased 30-day mortality and one year mortality was observed in patients with SBP < 100 mmHg (OR 1.9 [95% CI 1.4–2.4]) and SBP < 100 (OR 1.8 [1.2–2.6]) or EtCO2 < 4.0 kPa (OR 1.4 [1.1–1.5]), respectively. SpO2 was not significantly associated with either 30-day or one year mortality.

**Conclusions:**

After prehospital post-resuscitation stabilization, SBP < 100 mmHg and EtCO_2_ < 4.0 kPa were observed to be independently associated with higher mortality. The optimal targets for prehospital post-resuscitation care need to be established in the prospective studies.

## Introduction

International resuscitation guidelines describe post-resuscitation care as a vital factor in the chain of survival of patients suffering out-of-hospital cardiac arrest (OHCA). In the guidelines, post-resuscitation care is described primarily as hospital interventions and care.^1^, ^2^ Still, many patients who survive hospital admission die later in the intensive care unit (ICU) due to hypoxic-ischemic encephalopathy. Interventions in post-resuscitation care include targeted temperature management and early coronary angiography in selected patients.^3^, ^4^, ^5^, ^6^, ^7^ Furthermore, the goal-directed control of ventilation, oxygenation, and perfusion pressure to minimise the secondary consequence of hypoxic brain damage are used; however, the optimum goals for these controls are still under debate.^8^, ^9^, ^10^

Managing haemodynamics, ventilation and oxygenation are core competencies in prehospital critical care; many physician-staffed prehospital units frequently encounter patients resuscitated from OHCA.^11^, ^12^ Thus, goal-directed post-resuscitation care can be initiated in the very early reperfusion phase. However, most studies on physiological targets and outcomes after OHCA are conducted in the intensive care setting. In fact, to our knowledge, only two studies have explored the post-resuscitation oxygenation targets during the prehospital phase.^13^, ^14^ However, the significance and optimal targets of oxygenation, ventilation, and hemodynamic stabilization during the ultra-acute phase of reperfusion after return of spontaneous circulation may differ from later post-resuscitation intensive care.

We aimed to study the association among systolic blood pressure (SBP), peripheral oxygen saturation (SpO_2_) and end-tidal carbon dioxide (EtCO_2_) after prehospital stabilization to 30 day and one year survival. We hypothesised that the physiological range of these parameters, measured at the time of patient handover after prehospital stabilization, is associated with improved survival.

## Methods

### Study design

This retrospective registry-based study evaluated the association between the last vital signs measured in the prehospital phase after prehospital stabilization and survival in patients resuscitated from OHCA. The primary endpoint was 30 days all-cause mortality and secondary endpoint one-year all-cause mortality. Data collection was based on the national helicopter emergency medical services (HEMS) registry. The study – from January 2012 to August 2019 – was reported according to Strengthening the Reporting of Observational Studies in Epidemiology (STROBE) statement.^15^

The ethical committee of Helsinki University Hospital (HUS/3115/2019 §194) approved the study protocol. The hospital districts responsible for the HEMS (Oulu University Hospital 200/2019 2.7.2019, Helsinki University Hospital HUS/280/2019 9.7.2019, Turku University Hospital J30/19 4.8.2019, the Hospital District of Lapland 32/2019 22.8.2019, Kuopio University Hospital RPL 102/2019 22.8.2019 and Tampere University Hospital RTL-R19580 2.9.2019) and Population Register Centre (VRK/5613/2019–3 1.11.2019) also approved the protocol.

### Study setting

The Finnish HEMS, recently described in detail, is publicly funded and nationally organised in Finland.^12^ Medical responsibility lies in the hospital districts where the HEMS bases are. The HEMS covers most of the Finnish population of 5,5 million in 338,424 km.^12^ Five of the six HEMS units in Finland are staffed with physicians, primarily senior anaesthesiologists, and one by a specialised paramedic as well as the flight crew members.

The HEMS units are dispatched only for primary missions based on predefined criteria to support ground ambulances staffed by paramedics. The most common patient groups served by the HEMS are major trauma, unconsciousness and OHCA. The dispatching criteria include all witnessed OHCA cases when no do-not-attempt-resuscitation orders exist. For unwitnessed cases, HEMS is dispatched if the patient was seen alive within 20 minutes. The HEMS encounters approximately 1000 OHCA patients annually. Vasoactive drugs, advanced airway management and mechanical ventilation are frequently used in post-resuscitation care.^16^

### Study subjects

We included all OHCA patients treated by the HEMS who achieved a return of spontaneous circulation (ROSC) during prehospital care, were escorted by the HEMS team and survived to hospital admission. Unsuccessful or terminated resuscitation attempts or patients with unsustainable ROSC were not included. Patients were excluded if they lacked outcome data or data for all three of primary variables studied (SBP, SpO_2_, EtCO_2_).

There was no exclusion due to age or aetiology of cardiac arrest. Because of the heterogeneity of the OHCA patients, we also performed a subgroup analysis including only those with an initial shockable rhythm, presenting the more consistent group of patients with better outcomes.

### Variables

Every HEMS mission in Finland starting from 2012 have been recorded in the national database. The physician or advanced paramedic responsible for the treatment enters the data immediately after the mission. The database includes detailed data on the mission and the patient.^12^ The definitions in the database follow the guidelines on data collection and reporting on physician-staffed prehospital care, prehospital airway management and out-of-hospital cardiac arrest.^17^, ^18^, ^19^, ^20^

The following variables were retrieved from the HEMS database: patient demographics, initial rhythm, presumed aetiology of OHCA, bystander cardiopulmonary resuscitation (CPR), witness status of cardiac arrest, time from the emergency call to EMS and HEMS at the scene, delay from the emergency call to ROSC, vital signs (systolic blood pressure, peripheral oxygen saturation, end-tidal carbon dioxide) at the handover of the patient in the hospital. 30-day and 1-year mortality data were obtained from a population data services agency. Matching the data was based on a personal identification number (PIN) given to all citizens and residents. The data on survival was gathered in October 2019; thus, the latest patients did not receive a follow-up for one year.

### Statistical methods

Continuous variables are presented with medians and interquartile ranges (IQR) and compared using the Mann-Whitney U-test; categorical variables are presented with counts and percentages using the Chi-square test. We also visualised the risk of death for different values of vital signs by fitting locally estimated scatterplot smoothing (LOESS) curves for 30-day and one-year mortality.

Multivariable logistic regression models were fitted to investigate the association between 30-day and one-year mortality and patient vital signs. The degree of missingness in data is reported in Supplement 1. Reference values for post-resuscitation patient vital signs were SBP from 100 to 120 mmHg, SpO_2_ from 98 to 100% and EtCO_2_ from 4 to 4.5 kPa (30 to 34 mmHg). Reference values were chosen according to the guidelines during the study.^1^, ^2^ The multivariable logistic model was adjusted with gender, age, initial rhythm, bystander CPR and time interval from collapse to ROSC. The odds ratios (ORs) for the variables are shown using forest plots. We further investigated the multivariable models using mortality risk tables for different combinations of the SBP, SpO_2_ and EtCO2. In this model, only these vital signs were included and the model was not adjusted by other outcome-related variables due to limited sample size. The patients with missing variable necessary for the different models were excluded from these specific analyses. The missingness of the variables regarding the subgroup and outcome is presented in Supplement 1. The significance level was set to 0.05. All analyses were done with *stats* package using R version 4.0.3 (The R Foundation for Statistical Computing, Vienna, Austria) and the visualisation was done with the ggplot2 package. R package (A Language and Environment for Statistical Computing. The R Foundation for Statistical Computing, Vienna, Austria) was used in logistic regression modelling.

## Results

**Fig. 1:**
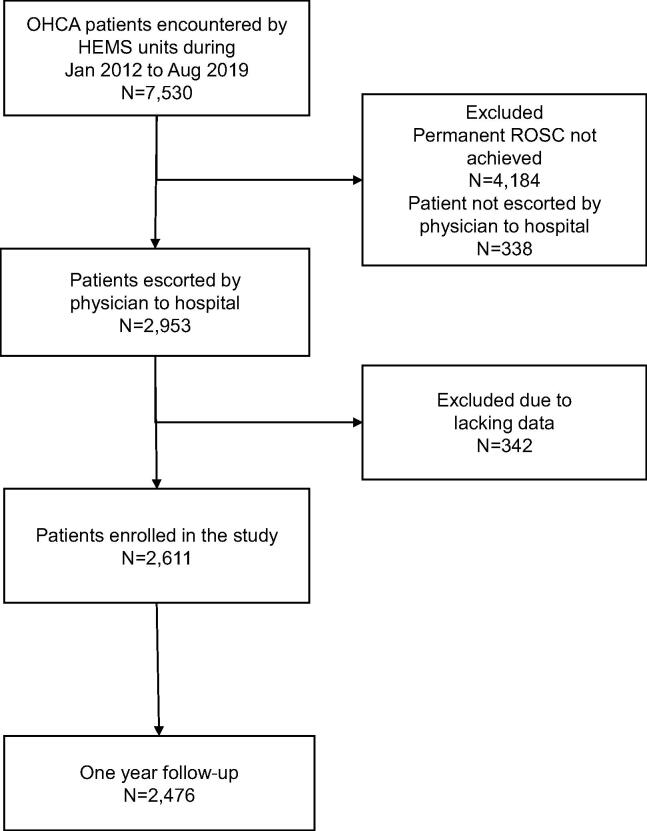
Patients selection flowchart. Patient enrolment in the study. HEMS, helicopter emergency medical services; OHCA, out-of-hospital cardiac arrest; ROSC, return of spontaneous circulation.

Of the 7530 OHCA patients the HEMS units encountered during the study, 2611 survived to hospital admission with spontaneous circulation and were included in the analyses (Fig. 1). The characteristics of the patients and cardiac arrests are in Table 1. The characteristics of the 342 patients excluded due to lacking data are detailed in Supplement 2. A comparison reveals that their demographics and cardiac arrest characteristics were similar to those of the included patients.

**Table 1:** Characteristics of the patients and cardiac arrests. Data is presented as a number (proportion) or median (interquartile range). Time intervals of EMS, HEMS and ROSC are presented from emergency calls. EMS, emergency medical services; HEMS, helicopter emergency medical services; CA, cardiac arrest.

	**All patients** **n = 2611**		**Patients with initial shockable rhythm** **n = 1412**	
Sex, male	1915	(73%)	1124	(79%)
Age, years	66	(56–74)	65	(56–73)
Witness status				
bystander-witnessed	2005	(77%)	1178	(83%)
unwitnessed	33	(1%)	18	(1%)
witnessed by EMS	573	(22%)	216	(15%)
Bystander CPR	1523	(58%)	949	(67%)
EMS delay, minutes	8	(6–12)	8	(6–11)
HEMS delay, minutes	24	(17–41)	23	(16–38)
Transportation, minutes	22	(12–38)	25	(13–40)
ROSC delay, minutes	20	(12–28)	20	(12–28)
Cause of CA				
Medical	1807	(69%)	1347	(95%)
Non-medical	804	(31%)	65	(5%)
Initial rhythm				
shockable	1412	(54%)	1412	(100%)
nonshockable	1199	(46%)	0	(0%)
Prehospital vasoactive medication administered	1846	(70%)	953	(67%)

The mortality data showed survival rate of 1,336/2,611 (51.2%) for primary endpoint of 30 days. For secondary endpoint, 1-year, 1071/2476 (43.3%) of the patients survived. The 30-day survival of patients with initial shockable rhythm was 987/1412 (69.9%); and for one-year survival was 825/1309 (63.0%).

**Fig. 2:**
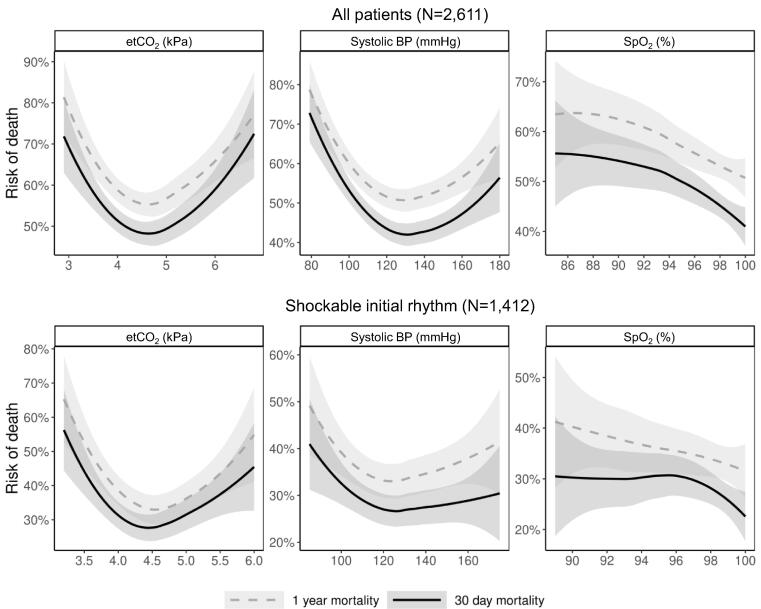
Estimated risk of death within 30 days and one year by last systolic blood pressure (BP), peripheral oxygen saturation (SpO_2_) and end-tidal partial pressure of CO_2_ (EtCO_2_) measured during prehospital care. Risk plots fitted using Loess methods. The variables were winsorized to 2.5 and 97.5 percentiles to avoid long uninformative tails in the plots.

Fig. 2 illustrates the LOESS-estimated percentual risk of death for different values of SBP, EtCO_2_ and SpO_2_. It is important to note that this representation is based on unadjusted data. In this unadjusted visualization, we observed that SBP values between 120–140 mmHg, EtCO_2_ levels of 4.0–4.5 kPa, and SpO2 of 100% were associated with the lowest risk of death.

**Fig. 3:**
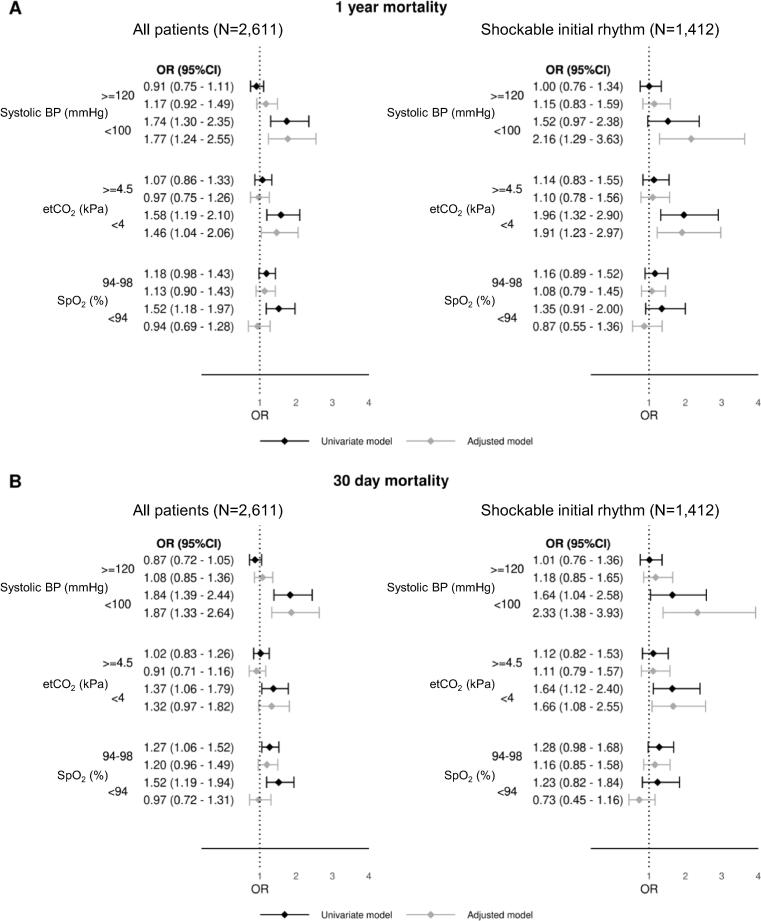
Logistic regression for risk of death within one year (A) and 30 days (B) by different categories of last systolic blood pressure (BP, n = 2,359 for 30 day mortality and n = 2,247 for 1 year mortality), peripheral oxygen saturation (SpO_2,_ n = 2,218 and 2,107) and end-tidal partial pressure of CO_2_ (EtCO_2_, n = 1,909 and 1,819) measured during prehospital care. Reference categories are systolic BP 100–119, EtCO2 4.0–4.4 and SpO2 99–100. Presented as odds ratio with 95% confidence interval. Adjusted model with age, sex, initial rhythm, ROSC delay, bystander CPR, witnessed status and cause of cardiac arrest.

The estimated risks of death by categorised SBP, EtCO_2_ and SpO_2_ are in Fig. 3. Increased 30-day mortality was observed in patients with SBP < 100 mmHg (OR 1.8 [95% CI 1.4 to 2.4]), EtCO_2_ < 4.0 kPa (OR 1.4 [95% CI 1.1 to 1.5]) and SpO2 < 94% (OR 1.5 [95% CI 1.2 to 1.9]). Increased one-year mortality was observed in patients with SBP < 100 mmHg (OR 1.7 [95% CI 1.3 to 2.4]), EtCO2 < 4.0 kPa (OR 1.6 [95% CI 1.2 to 2.1]), and SpO2 < 94% (OR 1.5 [95% CI 1.2 to 2.0]).

When adjusted with gender, age, initial rhythm, bystander CPR and time interval from collapse to ROSC, SBP < 100 mmHg (OR 1.87 [95% CI 1.33 to 2.64] and OR 1.77 [95% CI 1.24 to 2.55] for 30 days and one year, respectively) and EtCO_2_ < 4 kPa (OR 1.32 [95% CI 0.97 to 1.82] and OR 1.46 [95% 1.04 to 2.06] for 30 days and one year, respectively) were associated with an increase in mortality, whereas SpO_2_ < 94 was not associated with an increased risk of death (OR 0.97 [95% CI 0.72 to 1.31 and OR 0.94 [95% CI 0.69 to 1.28] for 30 days and one year, respectively).

**Fig. 4:**
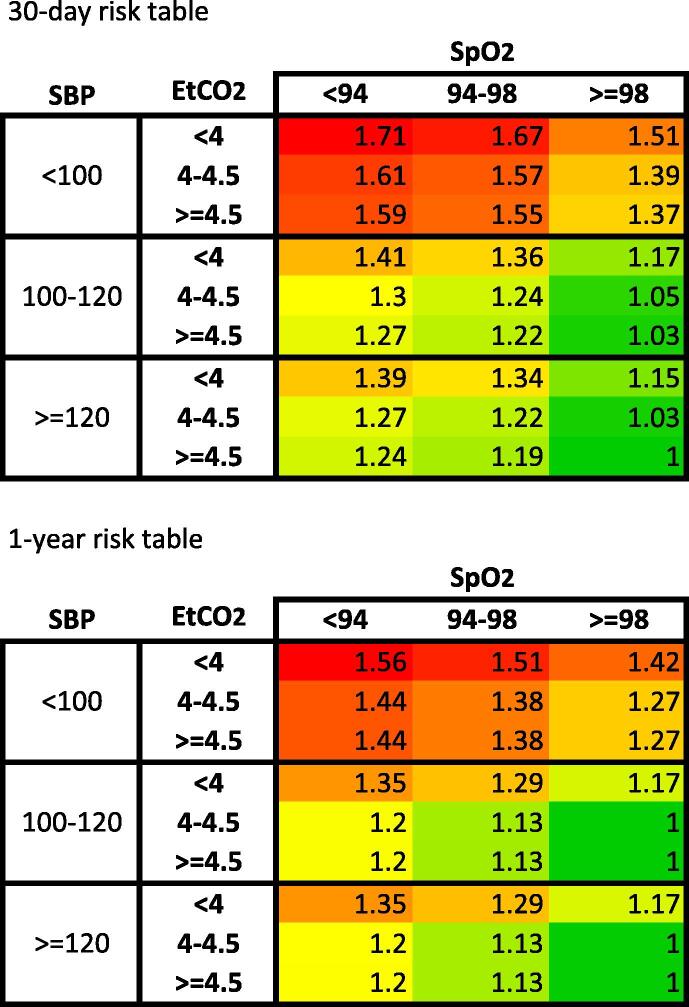
Cross-tabulation of relative risk of death in 30 days (A) and one year (B) in different combinations of systolic blood pressure (SBP), peripheral oxygen saturation (SpO_2_) and end-tidal partial pressure of carbon dioxide (EtCO_2_).

Cross-tabulation of relative risk of death by categorised levels of SBP, EtCO_2_ and SpO_2_ is presented in Fig. 4. Combination of SBP, EtCO_2_ and SpO2 with smallest risk for 30-day and one-year mortality was > 120 mmHg, >4.5 kPa, >98% and > 100 mmHg, > 4kPa and > 98%, respectively. The subgroup of patients with initial shockable rhythm contained similar findings (Supplement 3).

## Discussion

In this retrospective study of resuscitated OHCA patients, we found an association among hypocarbia and hypotension after preshospital post-resuscitation care and short- and long-term survival. The association was independent of known strong outcome predictors after cardiac arrest. The finding suggests that these parameters already in the “ultra-acute” post-resuscitation phase predicts long term outcome.

### Oxygenation in the early post-resuscitation care

Interestingly, our findings in unadjusted analyses peripheral oxygen saturation had no upper limit for a favourable outcome. While hypoxaemia (SpO_2_ < 94%) was associated with increased mortality risk in the unadjusted analyses, potential hyperoxaemia (SpO2 > 98%) after prehospital post-resuscitation care was not associated with increased mortality. However, in the adjusted analyses, hypoxaemia was not strong predictor of outcome nether. This notion is controversial to the current guidelines’ assumption. Cerebral hypoxaemia is associated with poor outcomes and contributes to developing a hypoxic-ischemic brain injury.^21^, ^22^ Also, early hyperoxaemia occuring one or two hours after ROSC has been demonstrated as associated with unfavourable outcomes in a study by Roberts et. al., including one with 280 patients.^23^ Later, various studies addressed hyperoxaemia during post-resuscitation care in the ICUs with mixed results.^24^, ^25^, ^26^, ^27^ The definitions of hyperoxaemia and assessment of its outcomes in these studies vary. In a randomised clinical trial, no negative association between moderate hyperoxaemia and six months of neurological outcome was observed when targeting moderate hyperoxaemia (20–25 kPa) by adjusting a fraction of the inspired oxygen and positive end-expiratory pressure (PEEP) for 36 hours after OHCA.^9^ This outcome was seen also in a recent RCT were patients were assigned either to a restrictive oxygen target (PaO_2_ of 9 to 10 kPa) or a liberal oxygen target (PaO_2_ of 13 to 14 kPa). The primary outcome of death or poor neurological survival at hospital discharge did not differ between these two groups.^28^ The potential tolerability or therapeutic effect of oxygen differs over time. Previous studies were mainly conducted in the ICU; researchers studied participants exposure to hyperoxaemia for several hours or even days. Henry et al.^29^ found that early hyperoxaemia (>40 kPa) happening less than two hours from hospital admission was not associated with increased hospital mortality. However, hyperoxaemia occurring later was associated with poor outcomes. This information aligns with our finding: Patients with higher SpO2 had lower 30-day mortality in unadjusted analyses. In our study, the median transportation time to hospital handover was 22 minutes. Such could explain the different findings between ours and previous studies and indicate that early hyperoxaemia in prehospital care is not necessarily harmful. However, comparisons with the earlier literature must be cautiously performed as we analysed only oxygen saturation measured with a pulse oximeter instead of arterial blood gases.

### End-tidal carbon dioxide in post-resuscitation care

Our study found that EtCO_2_ had a U-shaped curve for mortality, and the lowest risk was at approximately 4.5 kPa (34 mmHg). Further, hypocapnia (<4 kPa, 30 mmHg) and hypercapnia (>5kPa, 38 mmHg) were associated with increased mortality risk. In an adjusted analyse, only hypocapnia was significantly associated with poor outcome. Comparing the earlier studies is difficult as we analysed end-tidal values instead of arterial blood gases. The end-tidal partial pressure of carbon dioxide is affected by ventilation-perfusion matching and cardiac output, if they are significantly decreased. A considerable gap between EtCO_2_ and PCO_2_ in the anaesthetised patients in the prehospital setting has been recently demonstrated.^30^

In the observational ICU studies, patients with hypercapnia (PCO2 > 6 kPa) were more likely to be discharged home.^31^, ^32^ The randomised clinical trials found no improvement in the neurological outcome by high normal or mildly elevated PCO_2_.^9^, ^33^ Hypocapnia appears to impair neurological survival after cardiac arrest.^34^ Our prehospital findings align with these hospital-based studies showing that avoiding hypocapnia seems reasonable, whereas mild hypercapnia is unharmful.

### Blood pressure after cardiac arrest

Hypotension after cardiac arrest is common due to myocardial dysfunction from hypoxic-ischemic damage.^35^ Hypotension’s harmfulness and effect on outcome has been described in litterature.^36^, ^37^ This study implicates that SBP under 100 mmHg is harmful. Furthermore, we observed trend of better outcome in patients treated to SBP over 120 mmHg. However, because of the retrospective design, we cannot establish causation as we do not know whether the recorded blood pressure values were caused by pathophysiology, vasoactive drugs or other treatment.

Our results seem to agree with the recent studies conducted on ICU patients. Beylin et al.^37^ found that cardiac arrest survivors had higher mean arterial pressure (MAP) during the first 24 hours after ROSC than non-survivors. Interestingly, the mean MAP of the survivor cohort one hour after an arrest was 98 mmHg – significantly higher than currently recommended.^37^ Kilgannon et al.^38^ demonstrated a threshold of a MAP value greater than 70 mmHg as an independent factor for survival, with a mean MAP of 83 mmHg in the survivors. However, the randomised studies have shown no improvement in the neurological outcome by increasing the blood pressure over conventional targets.^8^, ^10^, ^39^ Our study demonstrates the association between survival and blood pressure value in the early phase after ROSC in the prehospital setting, whereas previous studies explored the blood pressure values hours after OHCA. Thus, comparing our results is challenging. Further, our study used SBP in our analyses while other studies used MAP. These two are not directly comparable and limits our study's generalization.

### Post-resuscitation care guidelines

According to the International Liaison Committee on Resuscitation (ILCOR)’s statement on post-resuscitation care, hypoxaemia, hyperoxaemia, and hypocapnia should be avoided.^40^

However, no specific numeric targets for post-resuscitation EtCO_2_ or PaCO_2_ are presented. Speaking of normocapnia is challenging since defining it is unclear. Depending on the source, normocapnia differs between 4.0 to 6.7 kPa.^41^ Furthermore, comparing EtCO_2_ values to paCO_2_values is challenging, although some models have been suggested.^42^

According to ILCOR’s statement, optimal blood pressure values associated with favourable neurological outcomes are unknown due to the low quality of evidence.^40^ The European Resuscitation Council (ERC) advises targeting systolic blood pressure over 100 mmHg – also used in the categorisation in our study.^1^ Since there is a lack of reliable data on the optimal blood pressure targets, we can only state that our study’s setting gave us a novel insight into the first minutes of early prehospital post-resuscitation care.

The optimal targets may differ during the different phases of post-resuscitation care, and the results of the ICU studies are not directly applicable in prehospital critical care. In the future, randomised studies should address the whole continuum of critical care, beginning in many systems already outside the hospital.

### Strengths and limitations

The national HEMS registry provides comprehensive, systematically collected, and high-quality data of numerous patients.^12,43^ However, our study has a few limitations:

Firstly, the study population is unsystematically selected as a physician can cancel HEMS involvement in the mission. Consequently, patients not considered to benefit from intensive care due to low functional capacity or difficult long-term diseases are not encountered. This evaluation was made without strict criteria and may lead to selection bias, reflected in the study’s relatively high survival rate.

Secondly, the treatment has not been standardised and can vary between cases and physicians. Although intubation is a standard treatment in our patient cohort, we cannot provide the exact number of patients intubated either during or after resuscitation. This limitation arises because our database only includes interventions by the HEMS units, and some patients may have been intubated by paramedics before the HEMS team's arrival.

Thirdly, study data were entered retrospectively and manually to the database. The entered data was not validated afterwards and is prone to self-reporting bias.

Finally, because of the study design and data sources used, the only outcome measured in the current study was mortality; causality between exposure and effect cannot be confirmed. Survival with sound neurological and functional conditions is the goal of treating OHCA patients, however in this study neurological survival could not be addressed. How much the prehospital interventions affected the exposures evaluated is unknown. A prospective intervention study is needed to establish causality.

## Conclusions

Blood pressure and ventilation after prehospital stabilization may be associated with the long-term outcome of patients resuscitated from OHCA. Hypocapnia (EtCO_2_ < 4.0) and hypotension (SBP < 100 mmHg) were associated with an increased risk of death, whereas oxygenation was not associated with risk of death. Randomised clinical trials, including the prehospital critical care phase, should be performed to ensure the exact targets for these parameters.

## CRediT authorship contribution statement

**Heikkilä Elina:** Conceptualization, Investigation, Methodology, Project administration, Writing – original draft, Writing – review & editing. **Setälä Piritta:** Conceptualization, Methodology, Supervision, Writing – original draft, Writing – review & editing. **Jousi Milla:** Conceptualization, Data curation, Formal analysis, Methodology, Project administration, Resources, Software, Supervision, Visualization, Writing – original draft, Writing – review & editing. **Nurmi Jouni:** Conceptualization, Data curation, Formal analysis, Methodology, Project administration, Resources, Software, Supervision, Visualization, Writing – original draft, Writing – review & editing.

## Declaration of competing interest

The authors declare that they have no known competing financial interests or personal relationships that could have appeared to influence the work reported in this paper.
