# Supplementary material for: Association among blood pressure, end-tidal carbon dioxide, peripheral oxygen saturation and mortality in prehospital post-resuscitation care
Source: Resusc Plus. 2024 Feb 13;17:100577. doi: 10.1016/j.resplu.2024.100577 (PMC10875297; doi:10.1016/j.resplu.2024.100577)
Supplement: Supplementary data 1 [file mmc1.docx]

**Supplement 1.** Level of missingness in the data.

|  | | | | | | |
| --- | --- | --- | --- | --- | --- | --- |
|  | 30d mortality | | | 1yr mortality | | |
| Variable | n_30d | survived_30d | death_30d | n_1yr | survived_1yr | death_1yr |
| Age | 104 (3.8%) | - | - | 239 (8.8%) | - | - |
| Blood pressure | 316 (11.6%) | 86 (27.2%) | 126 (39.9%) | 450 (16.6%) | 76 (16.9%) | 135 (30.0%) |
| Oxygen saturation | 458 (16.9%) | 128 (27.9%) | 226 (49.3%) | 590 (21.7%) | 109 (18.5%) | 242 (41.0%) |
| End-tidal CO_2_ | 776 (28.6%) | 423 (54.5%) | 249 (32.1%) | 881 (32.4%) | 346 (39.3%) | 296 (33.6%) |
| ROSC delay | 104 (3.8%) | - | - | 239 (8.8%) | - | - |
| Sex | 104 (3.8%) | 0 (0.0%) | 0 (0.0%) | 239 (8.8%) | 0 (0.0%) | 0 (0.0%) |
| Initial rhythm | 104 (3.8%) | 0 (0.0%) | 0 (0.0%) | 239 (8.8%) | 0 (0.0%) | 0 (0.0%) |
| Cause of arrest | 104 (3.8%) | 0 (0.0%) | 0 (0.0%) | 239 (8.8%) | 0 (0.0%) | 0 (0.0%) |
| Bystander CPR | 144 (5.3%) | 22 (15.3%) | 18 (12.5%) | 257 (9.5%) | 0 (0.0%) | 18 (7.0%) |
| Witness status | 104 (3.8%) | 0 (0.0%) | 0 (0.0%) | 239 (8.8%) | 0 (0.0%) | 0 (0.0%) |
