# Supplementary material for: Association among blood pressure, end-tidal carbon dioxide, peripheral oxygen saturation and mortality in prehospital post-resuscitation care
Source: Resusc Plus. 2024 Feb 13;17:100577. doi: 10.1016/j.resplu.2024.100577 (PMC10875297; doi:10.1016/j.resplu.2024.100577)
Supplement: Supplementary data 2 [file mmc2.docx]

**Supplement 2.** Characteristics of the patients excluded from the study due to lacking outcome data or lacking all the last measurements of systolic blood pressure, oxygen saturation and end-tidal carbon dioxide (n=343). Data are presented as n (percentage) or median (interquartile range).

| Sex, male | 197 | (57) |
| --- | --- | --- |
| Age, years | 64 | (54–74) |
| Witness status |  |  |
| bystander-witnessed | 256 | (75) |
| unwitnessed | 48 | (14) |
| witnessed by EMS | 39 | (11) |
| Bystander CPR | 228 | (66) |
| EMS delay, minutes | 8 | (6–12) |
| HEMS delay, minutes | 24 | (17–37) |
| Transportation, minutes | 23 | (15–135) |
| ROSC delay, minutes | 20 | (12–31) |
| Cause of CA |  |  |
| Medical | 239 | (70%) |
| Non-medical | 103 | (30%) |
| Initial rhythm |  |  |
| shockable | 191 | (56%) |
| non-shockable | 152 | (44%) |
| Prehospital vasoactive medication administered | 216 | (63%) |
